# Improving Glucocorticoid Sensitivity of Brain-Homing CD4^+^ T Helper Cells by Steroid Hormone Crosstalk

**DOI:** 10.3389/fimmu.2022.893702

**Published:** 2022-05-26

**Authors:** Steven C. Koetzier, Jamie van Langelaar, Annet F. Wierenga-Wolf, Marie-José Melief, Kim Pol, Suzanne Musters, Erik Lubberts, Willem A. Dik, Joost Smolders, Marvin M. van Luijn

**Affiliations:** ^1^ Department of Immunology, Erasmus Medical Center (MC), University Medical Center Rotterdam, Rotterdam, Netherlands; ^2^ Multiple Sclerosis (MS) Center ErasMS, Erasmus Medical Center (MC), University Medical Center Rotterdam, Rotterdam, Netherlands; ^3^ Department of Rheumatology, Erasmus Medical Center (MC), University Medical Center Rotterdam, Rotterdam, Netherlands; ^4^ Laboratory Medical Immunology, Erasmus Medical Center (MC), University Medical Center Rotterdam, Rotterdam, Netherlands; ^5^ Department of Neurology, Erasmus Medical Center (MC), University Medical Center Rotterdam, Rotterdam, Netherlands; ^6^ Neuroimmunology Research Group, Netherlands Institute for Neuroscience, Amsterdam, Netherlands

**Keywords:** calcitriol, progesterone, steroid resistance, T helper 17.1, multiple sclerosis

## Abstract

In early multiple sclerosis (MS), an IFN-γ^high^GM-CSF^high^IL-17^low^ CD4^+^ T-cell subset termed T helper 17.1 (Th17.1) reveals enhanced capacity to infiltrate the central nervous system. Th17.1 cells express high levels of multidrug resistance protein 1 (MDR1), which contributes to their poor glucocorticoid responsiveness. In this study, we explored whether glucocorticoid sensitivity of Th17.1 cells can generically be improved through synergy between steroid hormones, including calcitriol (1,25(OH)_2_D_3_), estradiol (E2) and progesterone (P4). We showed that human blood Th17.1 cells were less sensitive to 1,25(OH)_2_D_3_ than Th17 cells, as reflected by lower vitamin D receptor (*VDR*) levels and reduced modulation of MDR1, IFN-γ and GM-CSF expression after 1,25(OH)_2_D_3_ exposure. Upon T-cell activation, *VDR* levels were increased, but still lower in Th17.1 versus Th17 cells, which was accompanied by a 1,25(OH)_2_D_3_-mediated decline in MDR1 surface expression as well as secretion of IFN-γ and GM-CSF. In activated Th17.1 cells, 1,25(OH)_2_D_3_ amplified the suppressive effects of methylprednisolone (MP) on proliferation, MDR1 surface levels, secretion of IFN-γ and granzyme B, as well as expression of brain-homing markers CCR6 and VLA-4. The addition of P4 to 1,25(OH)_2_D_3_ further enhanced MP-mediated reduction in proliferation, CD25, CCR6 and CXCR3. Overall, this study indicates that glucocorticoid sensitivity of Th17.1 cells can be enhanced by treatment with 1,25(OH)_2_D_3_ and further improved with P4. Our observations implicate steroid hormone crosstalk as a therapeutic avenue in Th17.1-associated inflammatory diseases including MS.

## Introduction

Multiple sclerosis (MS) is a chronic inflammatory disease of the central nervous system (CNS) (1). In the early phases of the disease, attacks of MS are treated with high dosages of pulsed synthetic glucocorticoids such as methylprednisolone (MP) (2). Although this treatment shortens attacks, it does not improve the level of recovery (3–5) and is associated with various adverse effects (6, 7). Remarkably, the glucocorticoid sensitivity of white blood cells during MS disease progression is decreased (8). Since the mechanism of MP action relies on the chemokine-dependent redirection of CNS-infiltrating T cells in EAE (9, 10), this raises the question whether CNS-homing T cells in humans are selectively able to circumvent this drug and thus determine the longevity of this treatment in MS.

Previously, we showed that a chemokine receptor-defined CD4^+^ T-cell subset termed T helper 17.1 (Th17.1; CCR6^+^CXCR3^+^CCR4^-/dim^) is associated with early MS activity (11). This subset is not only characterized by the expression of IFN-γ, GM-CSF and granzyme B, but is also refractory to glucocorticoids due to the abundance of surface multidrug resistance receptor 1 (MDR1) expression (11–13). Th17.1 cells were absent in the peripheral blood of early MS patients and selectively targeted by natalizumab, an anti-VLA4 antibody that prevents immune cells from entering the CNS and allows for their selective accumulation in the circulation (11). Accordingly, Th17.1 cells dominated the cerebrospinal fluid (CSF) of treatment-naive early MS patients and were present in MS brain white matter lesions (12). Intriguingly, the effector function of these cells seemed to be controlled during pregnancy and related to a postpartum relapse (14), indicating that female hormones can suppress the pathogenicity of Th17.1 cells.

Functional studies have shown that calcitriol (1,25(OH)_2_D_3_), the active metabolite of vitamin D_3_, enhances MP action *in vitro* (15), but vitamin D_3_ supplementation showed limited to no clinical benefits in MS trials (16). Interestingly, vitamin D_3_ was able to ameliorate disease activity in EAE (17, 18), which depended on the presence of female hormones (19–21).

In this study, we hypothesized that MP responsiveness of human Th17.1 cells can be optimized through crosstalk between 1,25(OH)_2_D_3_ and female hormones. Since these cells are absent in the circulation of MS patients (11), and steroid resistance is a generic trait of these cells (12), we primarily utilized Th17.1 from healthy blood donors for the current experiments. We first investigated the sensitivity of blood Th17.1 and Th17 cells to 1,25(OH)_2_D_3_ and how this is influenced upon T-cell receptor (TCR) activation. Next, we assessed whether 1,25(OH)_2_D_3_ and further addition of pregnancy-related dosages of estradiol (E2) and progesterone (P4) could enhance MP-mediated suppression of activated Th17.1 cells *in vitro*.

## Materials and Methods

### Sampling and Ethics

Healthy donor peripheral blood mononuclear cells (PBMCs) were collected using CPT tubes (BD Biosciences, Erembodegem, Belgium) containing sodium heparin for cell-based analysis and isolated according to manufacturer’s instructions. PBMCs were frozen down in RPMI 1640 with L-Glutamine (Lonza, Verviers, Belgium) containing 20% fetal calf serum (Thermo Fisher Scientific, Landsmeer, The Netherlands) and 10% dimethyl sulfoxide (Sigma-Aldrich, St Louis, MO, USA), and stored in liquid nitrogen until further use. MS patients were diagnosed based on the McDonald 2017 criteria and included at the MS center ErasMS, Erasmus MC. Blood samples were collected from MS patients treated with natalizumab for 18 months. The studies involving human participants were reviewed and approved by Medical Ethics Committee Erasmus MC (MEC-2014-033). Cohort characteristics are summarized in **Supplementary Table 1**.

### Flow Cytometry and Cell Sorting

The fluorescently labeled anti-human monoclonal antibodies used for this study are shown in **Supplementary Table 2**. Surface markers were stained for 30 min at 4°C in the dark. When applicable, prior to each staining, cells were incubated with anti-MDR1 antibody in RPMI 1640 containing 2% fetal calf serum and 25 µM cyclosporine A (Sigma-Aldrich) for 20 min at 37°C. For exclusion of dead cells, Fixable Viability Stain 700 (BD Biosciences) was added for 15 min at 4°C in the dark. Cells were measured using an LSRII-Fortessa (BD Biosciences) and analyzed using BD FACSDiva (version 8.0.1) software. Memory CD4^+^ T cells were isolated from fresh healthy donor blood (Sanquin, Amsterdam, The Netherlands) using the human Memory CD4^+^ T cell Isolation Kit and the autoMACS Pro Separator (both Miltenyi Biotec, Bergisch Gladbach, Germany) and frozen down as described above. Memory CD4^+^ (CD45RA^-^CD25^low/-^) T-cell subsets Th17 (CCR6^+^CXCR3^-^CCR4^+^) and Th17.1 (CCR6^+^CXCR3^+^CCR4^-/dim^) were purified using a FACSAria-III machine (BD Biosciences).

### RNA Isolation and Quantitative PCR

Th17 and Th17.1 cells were either used unstimulated or plated at 0.5x10^6^/ml in RPMI 1640 containing 5% inactivated human AB serum (Sanquin), 100 U/ml penicillin (Pen) and 100 μg/ml Streptomycin (Strep) at 37°C. After sorting, cells were directly lysed or stimulated with aCD3/CD28 dynabeads (1:5; Thermo Fisher Scientific) for 24 h and/or stimulated with phorbol 12-myristate 13-acetate (PMA; 50 ng/ml) and ionomycin (1 µg/ml; both Sigma-Aldrich) for 5 h the following day. In case of 1,25(OH)_2_D_3_ (0.1 µM; Sigma-Aldrich) stimulations, cells were plated at a concentration of 1.25×10^5^/ml in the same medium. RNA isolation, complementary DNA synthesis, and real-time quantitative PCRs were performed as previously described (11). Primer-probe sets were designed using the Universal ProbeLibrary (Roche Applied Science, Penzberg, Germany) and primer sequences are displayed in **Supplementary Table 3**.

### *In Vitro* Proliferation Assay

Healthy donor memory CD4^+^ T cells were stained with 0.075 µM CellTrace Carboxyfluorescein Succinimidyl Ester (CFSE) according to manufacturer’s instructions (Thermo Fisher Scientific). After staining, Th17.1 cells were sorted, plated at 1.25 × 10^5^ cells/mL and activated with aCD3/CD28 dynabeads (1:5; Thermo Fisher Scientific) for 72 h. Cells were simultaneously cultured in RPMI 1640 supplemented with 100 U/ml Pen and 100 μg/ml Strep, 5% human AB serum (Sanquin), and a patient-relevant dosage (15) of 75 µM MP (Pfizer, Capelle a/d IJssel, The Netherlands) and/or different combinations of 0.1 µM 1,25(OH)_2_D_3_ (Sigma-Aldrich), 0.1 µM water-soluble E2 (Sigma-Aldrich), 2 µg/ml γ-irradiated P4 (Sigma-Aldrich), in addition to the appropriate vehicle controls. Finally, cells were washed and stained for flow cytometry as described. After 72 h, supernatants were collected and stored at -80°C until further use. Values of the treatment conditions were divided against the relevant vehicle controls and converted into percentages. These values were subtracted from 100 to depict the percentage of suppression.

### Cytokine Measurement

Culture supernatants were diluted twofold and analyzed for GM-CSF, granzyme B, IFN-γ, IL-10, tumor necrosis factor alpha (TNF-α) and lymphotoxin-α (LT) levels using a custom Luminex multiplex bead immunoassay (R&D Systems, add City, UK). Measurements were performed on a Bio‐Plex MAGPIX machine and data were analyzed using Bio‐Plex Manager MP software (both Bio-Rad, Hercules, California, USA).

### Statistics

Statistics were performed using GraphPad Prism 9 software and are described in detail within each figure legend. Data are displayed as individual data points with or without the standard error of the mean. For all tests, a *P* value of < 0.05 (*) was considered significant.

## Results

### Reduced Sensitivity of Glucocorticoid-Resistant Th17.1 Cells to Calcitriol Is Enhanced Upon Activation

**Figure 1:**
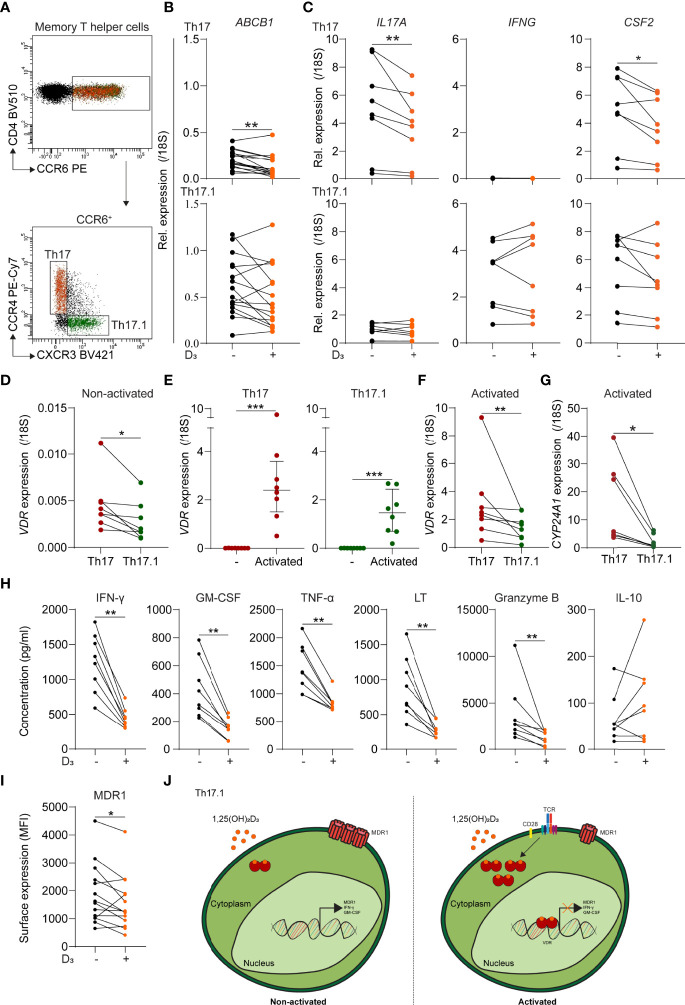
The sensitivity of MDR1^low^ Th17 and MDR1^high^ Th17.1 cells to 1,25(OH)_2_D_3._ **(A)** FACS plot showing representative gating for CCR6^+^ memory T helper (CD4^+^CD45RA^-^CD25^low^) subsets Th17 (CCR4^+^CXCR3^-^) and Th17.1 (CCR4^-/dim^). **(B)** Relative *ABCB1* expression for healthy donor Th17 and Th17.1 cells as determined by qPCR (*n* = 16 per group). **(C)** Relative *IL-17A, IFNG and CSF2* expression for healthy donor PMAionomycin and anti-CD3/CD28-stimulated Th17 and Th17.1 cells before and after 1,25(OH)_2_D_3_ exposure as determined by qPCR (*n* = 7 per group). **(D)** Relative *VDR* expression for healthy donor non-activated Th17 and Th17.1 cells as determined by qPCR (*n* = 7 per group). **(E)** Relative *VDR* expression for healthy donor non-activated versus anti-CD3/CD28 activated Th17 and Th17.1 cells, including a comparison for these activated Th17 and Th17.1 cells **(F)**, as determined by qPCR (*n* = 8 per group). **(G)** Relative *CYP24A1* expression for healthy donor PMAionomycin and anti-CD3/CD28-stimulated Th17 and Th17.1 cells after 1,25(OH)_2_D_3_ exposure as determined by qPCR (*n* = 7 per group). **(H)** Amount (pg/ml) of IFN-γ, GM-CSF, TNF-α, LT, granzyme B, and IL-10 measured in the supernatants of healthy donor anti-CD3/CD28-stimulated Th17.1 cells before and after 1,25(OH)_2_D_3_ exposure as determined by Luminex (*n* = 8 per group). **(I)** MDR1 surface expression (median fluorescent intensity) on healthy donor anti-CD3/CD28-stimulated Th17.1 cells before and after 1,25(OH)_2_D_3_ exposure (*n* = 13 per group). **(J)** Graphical model displaying the 1,25(OH)_2_D_3_ sensitivity of non-activated versus activated Th17.1 cells. When TCR-activated, Th17.1 cells increase their VDR expression resulting in 1,25(OH)_2_D_3_-signaling and a decrease in their pro-inflammatory (IFN-γ^+^GM-CSF^+^) and MDR1-expressing phenotype. Lines represent paired observations for cells from the same donors. Data were compared using either Wilcoxon rank-sum or **(E)** Mann-Whitney U tests. *p < 0.05, **p < 0.01 and ***p < 0.001. “D_3_ = 1,25(OH)_2_D_3_”, “MDR1”, multidrug resistance protein 1; “MFI, median fluorescence intensity”; “1,25(OH)_2_D_3_, calcitriol”, “TCR”, T-cell receptor and “VDR, vitamin D receptor”.

In contrast to paired MDR1^low^ Th17 cells (*p* < 0.01), *ex vivo* MDR1^high^ Th17.1 cells from healthy donors showed no reduction in *ABCB1* (MDR1) levels after 24 h stimulation with 1,25(OH)_2_D_3_ (**Figures 1A, B**) (12). Additionally, the relative expression of Th17-associated *IL17A* (IL-17A) and *CFS2* (GM-CSF) was decreased (*p* < 0.05 and 0.01), while this was not the case for Th17.1-associated *IFNG* (IFN-γ) and *CSF2* (**Figure 1C**). *Vitamin D receptor* (*VDR*) levels were significantly lower in resting Th17.1 compared to Th17 cells (**Figure 1D**; *p* < 0.05), supporting their reduced sensitivity to 1,25(OH)_2_D_3_. Because vitamin D_3_ effects are induced upon T-cell activation (22), we stimulated purified Th17.1 and Th17 cells with anti-CD3/CD28 for 24 h. *VDR* levels were increased in both subsets (**Figure 1E**, p < 0.001), but remained higher in Th17 versus Th17.1 cells (**Figure 1F**; *p* < 0.01). This was consistent with the reduced VDR signaling observed in Th17.1 compared to Th17 cells, reflected by the lower expression of *Cytochrome P450 family 24 subfamily A member 1* (*CYP24A1*) after exposure to 1,25(OH)_2_D_3_ (**Figure 1G**; *p* < 0.05) (23). Nonetheless, the activation-induced upregulation of *VDR* did allow us to study whether 1,25(OH)_2_D_3_ has the potential to modulate the pathogenicity of Th17.1 cells. Upon activation, 1,25(OH)_2_D_3_ attenuated the excretion of Th17.1-associated IFN-γ and GM-CSF, as well as TNF-α, LT and granzyme B (**Figure 1H**; all *p* < 0.01). No changes were observed for IL-10 (**Figure 1H**). Moreover, MDR1 surface expression was lowered after stimulation of activated Th17.1 cells with 1,25(OH)_2_D_3_ (**Figure 1I**; *p* < 0.01).

These results indicate that *ex vivo* Th17.1 cells are relatively insensitive to 1,25(OH)_2_D_3_, which potentially contributes to their pro-inflammatory and glucocorticoid-resistant phenotype (**Figure 1J**). However, following activation, 1,25(OH)_2_D_3_ seems to be useful to reduce MDR1 expression and thereby increase glucocorticoid responsiveness. Previously, we showed no difference in *VDR* and *CYP24A1* expression between CD4^+^ T cells of MS and control donors (24), and others showed no differences between MS and control donors in suppression of CD4^+^ T cell proliferation and cytokine production by 1,25(OH)_2_D (25). We currently expanded these data by showing no difference in *VDR* expression between control and natalizumab-treated MS Th17 and Th17.1 cells (**Supplementary Figure 1**). Therefore, we assume similar vitamin D responsiveness between Th17.1 cells of MS and control donors.

### Calcitriol and Progesterone Optimally Sensitize Th17.1 Cells to Methylprednisolone

**Figure 2:**
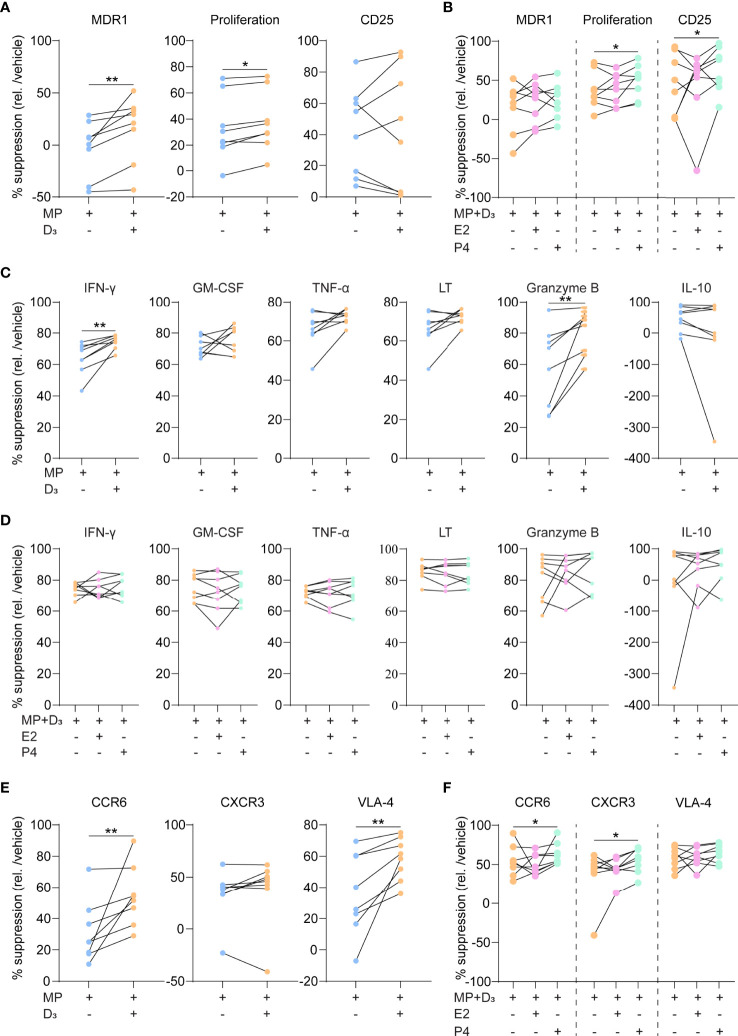
The suppressive capacity of steroid hormone cocktails on glucocorticoid-resistant Th17.1 cells. **(A)** MDR1 expression, proliferation rates (CFSE^-^) and CD25 surface expression (MFI) by healthy donor anti-CD3/CD28-stimulated Th17.1 cells exposed to MP with and without D_3_ as determined by flow cytometry (*n* = 8). Percentages are relative to their appropriate vehicle control. **(B)** The same parameters as in A) for healthy donor anti-CD3/CD28-stimulated Th17.1 cells exposed to MP+D_3_ with and without E2 or P4 as determined by flow cytometry (*n* = 8). Percentages are relative to their appropriate vehicle control. **(C)** Amount (pg/ml) of IFN-γ, GM-CSF, TNF-α, LT, granzyme B, and IL-10 measured in the supernatants of healthy donor anti-CD3/CD28-stimulated Th17.1 cells exposed to MP with and without D_3_ as determined by Luminex (*n* = 8 per group). **(D)** Amount (pg/ml) of IFN-γ, GM-CSF, TNF-α, LT, granzyme B, and IL-10 measured in the supernatants of healthy donor anti-CD3/CD28-stimulated Th17.1 cells exposed to MP+D_3_ with and without E2 or P4 as determined by Luminex (*n* = 8 per group). **(E)** CCR6, CXCR3 and VLA-4 surface expression on healthy donor anti-CD3/CD28-stimulated Th17.1 cells exposed to MP with and without D_3_ as determined by flow cytometry (*n* = 8). Percentages are relative to their appropriate vehicle control. **(F)** The same parameters (as in E) for healthy donor anti-CD3/CD28-stimulated Th17.1 cells exposed to MP+D_3_ with and without E2 or P4 as determined by flow cytometry (*n* = 8). Percentages are relative to their appropriate vehicle control. Data were compared using either Wilcoxon rank-sum or **(D)** Friedman tests with the false discovery rate of Benjamini, Krieger and Yekutieli correction. *p < 0.05 and **p < 0.01. “D_3_ = 1,25(OH)_2_D_3_”, “E2, Estradiol”; “MFI, median fluorescence intensity”; “MP, methylprednisolone” and “P4, progesterone”.

To address whether 1,25(OH)_2_D_3_ potentiates their response to glucocorticoids, we added MP to purified, activated Th17.1 cells for 3 days with and without 1,25(OH)_2_D_3_. Since female hormones are able to increase 1,25(OH)_2_D_3_ sensitivity (21), we also investigated whether there was an enhanced effect when pregnancy-related dosages of E2 or P4 were added. Both MDR1 surface expression and CFSE-based cell proliferation were more suppressed by MP in the presence of 1,25(OH)_2_D_3_ (**Figure 2A**; *p* = 0.01 and 0.05, respectively). 1,25(OH)_2_D_3_ did not induce MP-mediated reduction in CD25 surface levels (**Figure 2A**). The addition of P4 to 1,25(OH)_2_D_3_ and MP further reduced proliferation rates and resulted in a downregulation of CD25 (**Figure 2B**; both *p* < 0.05). This was not seen when using E2 (**Figure 2B**). 1,25(OH)_2_D_3_ also induced MP-mediated suppression of IFN-γ and granzyme B excretion (**Figure 2C**; both *p* < 0.05), which was not potentiated by P4 and E2 (**Figure 2D**). The inhibitory effects of MP on GM-CSF, TNF-α, LT and IL-10 excretion were not affected by 1,25(OH)_2_D_3_, P4 and E2 (**Figures 2C, D**). Finally, 1,25(OH)_2_D_3_ enhanced the MP-induced downregulation of surface CCR6 and VLA-4 (both *p* < 0.01), while surface CXCR3 expression was not affected (**Figure 2E**). In contrast to E2, P4 further reduced CCR6 and additionally lowered CXCR3 levels (both *p* < 0.05), which was not seen for VLA-4 (**Figure 2F**).

**Figure 3:**
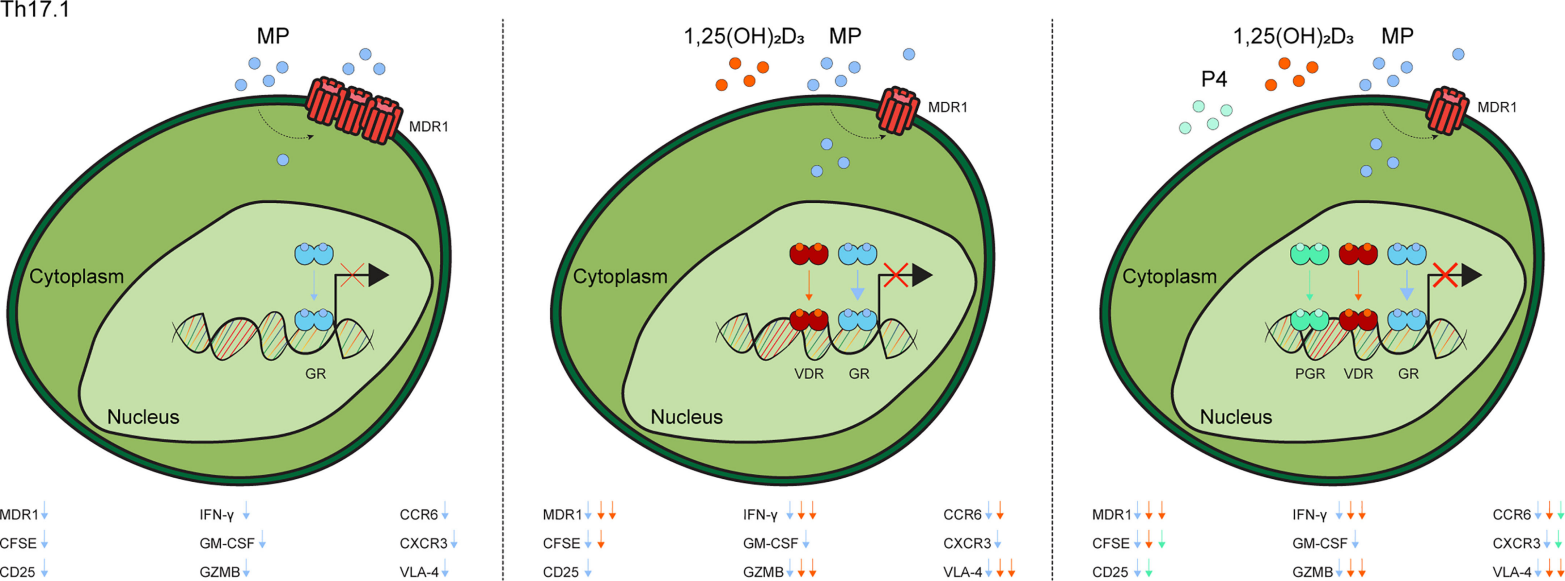
Graphical model of the additive value of 1,25(OH)_2_D_3_ and progesterone on methylprednisolone-induced suppression of Th17.1 cells. In contrast to when Th17.1 are only exposed to MP, treatment with 1,25(OH)_2_D_3_ lowers their MDR1 expression and thus increases GR-signaling, thereby providing some or optimal inhibition of pathogenic markers. Addition of P4 leads provides additional suppression of some pathogenic markers. The exact modulated markers are given in each subfigure. “1,25(OH)_2_D_3_ = calcitriol”, “CFSE”, Carboxyfluorescein Succinimidyl Ester; “MDR1”, multidrug resistance protein 1; “MP”, methylprednisolone”; “GR, glucocorticoid receptor”; “GZMB”, granzyme B; “P4, progesterone” and “PGR, progesterone receptor” and “VDR, vitamin D receptor”.

These data indicate that 1,25(OH)_2_D_3_ and P4 optimize MP-mediated suppression of Th17.1 cells, as reflected by steroid-dependent reductions in MDR1 levels, proliferative capacity and both pro-inflammatory and brain-homing markers (**Figure 3**).

## Discussion

Synthetic glucocorticoids are often used to shorten clinical symptoms in chronic inflammatory diseases, including MS (2). However, a proportion of MS patients develop glucocorticoid resistance during disease progression (8), long term prognosis and recovery are not affected, and usage of this drug associates with putative side effects (6). Further improvement of glucocorticoid responses could therefore be of benefit in these patients, which makes it crucial to understand and modulate the underlying mechanism of resistance in immune subsets. Previously, we identified that Th17.1 cells are refractory to glucocorticoids, corresponding to their pro-inflammatory capacity and selective recruitment to the CNS of MS patients (12). In this study, we provide evidence that Th17.1 cells can be sensitized to MP using 1,25(OH)_2_D_3_ in a process that is further potentiated by the addition of P4.

We found that 1,25(OH)_2_D_3_ treatment decreased MDR1 gene (*ABCB1*) expression in resting Th17, but not in Th17.1 cells, which is consistent with a study showing that *ABCB1* contains a VDRE in its promotor region (26). Furthermore, others demonstrated that 1,25(OH)_2_D_3_ can suppress *IL17A*, *CSF2* and *IFNG* levels in CCR6^+^ Th cells (27). We now demonstrate that such effects are different between CCR6^+^ Th subsets, with Th17 cells being more sensitive to 1,25(OH)_2_D_3_ than Th17.1 cells. This impaired sensitivity of Th17.1 cells was also reflected by their lower *VDR* and *CYP24A1* levels. Besides for pro-inflammatory cytokines, the expression of other known hallmarks for 1,25(OH)_2_D_3_ responses such as forkhead box P3 and apoptosis-associated genes should be assessed to validate these results (28). Given the fact that Th17.1 and not Th17 cells preferentially infiltrate the MS brain (12), this makes it tempting to speculate that the limited success of vitamin D_3_ in MS clinical trials (16) is at least partially due to the insensitivity of such immune subsets to 1,25(OH)_2_D_3_.

In addition, *VDR* was upregulated in Th17.1 cells upon activation, which is in line with earlier findings in T cells (29). Under these conditions, 1,25(OH)_2_D_3_ treatment decreased IFN-γ, GM-CSF, TNF-α, LT and granzyme B excretion as well as MDR1 expression by Th17.1 cells. Together with the association of activated Th17.1 cells (11, 14) and low circulating 25(OH)D levels (30) with MS relapses, one can generate a hypothesis in which there is a therapeutic window of opportunity for vitamin D_3_ supplementation, also in relation to improving glucocorticoid responses, in the earliest relapsing phases of MS. As *VDR* expression was not different between Th17.1 cells from healthy controls and natalizumab-treated MS patients it can be expected that there are no differences in the responses of these cells to 1,25(OH_2_)D_3_ treatment as was shown previously for bulk CD4^+^ T cells (25). Nevertheless, MS-specific *in vivo* differences in vitamin D responsiveness of Th17.1 cells could be present. We cannot exclude that *VDR* expression levels were influenced by natalizumab. In addition, MS-associated single nucleotide polymorphisms in both *VDR* (31) and *CYP24A1* (32) genes have been described, which also affect responses to vitamin D_3_ supplementation in MS (33). Therefore, these factors should be accounted for when further addressing this hypothesis.

Cell proliferation and migration are important target mechanisms of glucocorticoids. Previously, we confirmed that MDR1^high^ Th17.1 cells are relatively glucocorticoid resistant when compared to MDR1^low^ Th17 cells in the context of MS (12). After TCR activation, 1,25(OH)_2_D_3_ co-treatment enhanced MP-mediated suppression of Th17.1 cell proliferation. Although this is likely due to downregulation of surface MDR1 expression, it is also known that 1,25(OH)_2_D_3_ by itself can limit proliferation of T cells (34). Interestingly, P4 supplementation further inhibited the proliferation of Th17.1 cells, which was accompanied by a reduction in CD25 expression. These effects are probably interrelated given the impact of autocrine IL-2 signaling on T-cell proliferation in relation to MS (35, 36). 1,25(OH)_2_D_3_ also increased the MP-induced suppression of IFN-γ and granzyme B, which was not potentiated by co-treatment with P4 or E2. MP impaired the expression of CCR6, VLA-4 and CXCR3 by Th17.1 cells, of which CCR6 and VLA-4 were further reduced after co-treatment with 1,25(OH)_2_D_3_. This is in line with other studies showing that 1,25(OH)_2_D_3_ modulates CCR6 (27) and VLA-4 (37), but not CXCR3 expression (27). P4 co-supplementation lowered CCR6 levels even more and showed a reducing effect on CXCR3. This strongly implies that the brain-homing potential of Th17.1 cells is selectively reduced through synergy between MP, 1,25(OH)_2_D_3_ and P4. Efficient suppression of such brain-homing markers is warranted to prevent CCR6-, CXCR3- and- VLA-4-mediated transmigration of pathogenic T cells across the choroid plexus (38) and the blood-brain barrier (39, 40) respectively. Furthermore, CXCL10 is highly enriched in MS CSF (41), indicating that additional targeting of CXCR3 could be crucial to completely suppress the brain-homing capability of Th17.1 cells. Using cocktails of steroid hormones for this purpose is further supported by the fact that 1,25(OH)_2_D_3_-treated CCR6^+^ Th cells are still able to migrate towards CXCL10 *in vitro* (27). Our results indicate that especially P4 supplementation can increase the efficiency of MP and 1,25(OH)_2_D_3_ co-treatment. Consistently, P4 was found to directly upregulate VDR expression and suppress human T cells (42, 43). Lastly, it should be assessed whether these observations are due to direct nuclear receptor-target gene interaction or due to secondary responses. This would also be of benefit to exclude to possibility of adverse effects.

Overall, co-supplementation of MP and vitamin D, eventually further potentiated with P4, may optimize MP responses in MS patients *via* the suppression of pathogenic Th17.1 cells. Since Th17.1 cells are key drivers of MS activity (11, 12, 14), this optimal suppression may induce not only a swifter but also a better recovery and more long-term protection in these patients.

## Data Availability Statement

The original contributions presented in the study are included in the article/**Supplementary Material**. Further inquiries can be directed to the corresponding author.

## Ethics Statement

This study involved human participants and was reviewed and approved by Medical Ethics Committee Erasmus MC. The participants provided their written informed consent to participate in this study.

## Author Contributions

SK performed experiments, analyzed data, interpreted results, and wrote the manuscript. JL assisted with the study concept, interpretation of the results and critically revised the manuscript. AW-W and M-JM performed experiments. EL and WD analyzed data and critically revised the manuscript. JS and ML designed the research, obtained funding, interpreted results, and critically revised the manuscript. All authors contributed to the article and approved the submitted version.

## Funding

This study was funded by the Dutch MS Research Foundation (15-490d MS, 16-952 MS and 20-490f MS).

## Conflict of Interest

ML received research support from EMD Serono, GSK en Idorsia Pharmaceuticals Ltd. JS received lecture and/or consultancy fee from Biogen, Merck, Novartis, and Sanofi-Genzyme.

The remaining authors declare that the research was conducted in the absence of any commercial or financial relationships that could be construed as a potential conflict of interest.

## Publisher’s Note

All claims expressed in this article are solely those of the authors and do not necessarily represent those of their affiliated organizations, or those of the publisher, the editors and the reviewers. Any product that may be evaluated in this article, or claim that may be made by its manufacturer, is not guaranteed or endorsed by the publisher.
