# Supplementary material for: Improving Glucocorticoid Sensitivity of Brain-Homing CD4+ T Helper Cells by Steroid Hormone Crosstalk
Source: Front Immunol. 2022 May 26;13:893702. doi: 10.3389/fimmu.2022.893702 (PMC9178273; doi:10.3389/fimmu.2022.893702)
Supplement: Supplementary file 1 [file DataSheet_1.pdf]

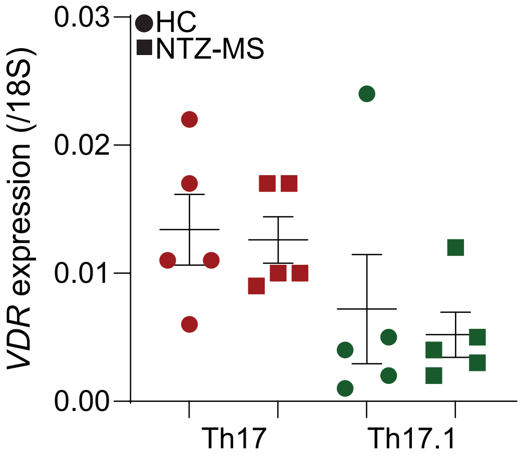

Supplementary Figure 1. *VDR* expression by healthy- and MS-derived Th17 and Th17.1 cells. Relative *VDR* expression by unstimulated Th17 and Th17.1 cells from healthy controls (HC, *n* = 5) and MS patients treated with natalizumab for 12 months (NTZ-MS, *n* = 5). Data were compared using Mann-Whitney U tests.
