# Supplementary material for: Improving Glucocorticoid Sensitivity of Brain-Homing CD4+ T Helper Cells by Steroid Hormone Crosstalk
Source: Front Immunol. 2022 May 26;13:893702. doi: 10.3389/fimmu.2022.893702 (PMC9178273; doi:10.3389/fimmu.2022.893702)
Supplement: Supplementary file 2 [file Table_1.docx]

| **qPCR** | *ABCB1* | *IL17A IFNG CSF2* | Non-activated *VDR* | Activated *VDR* | *CYP24A1* |
| --- | --- | --- | --- | --- | --- |
| N (% female) | 16 (43) | 10 (60) | 8 (63) | 8 (50) | 7 (57) |
| Median age in years (min-max) | 54 (27-67) | 55 (27-63) | 52 (24-65) | 53 (25-65) | 47 (27-63) |
| **Luminex** | 1,25(OH_2_)D_3_ | Cocktails | **FACS** | 1,25(OH_2_)D_3_ | Cocktails |
| N (% female) | 8 (63) | 8 (63) |  | 14 (57) | 8 (63) |
| Median age in years (min-max) | 33 (27-65) | 33 (27-65) |  | 46 (27-65) | 33 (27-65) |

**Supplementary Table 1a:** Cohort characteristics for healthy controls

**Supplementary Table 1b:** Cohort characteristics for healthy control versus natalizumab-treated MS qPCRs

| ***VDR*** | Healthy controls | Natalizumab-treated MS |
| --- | --- | --- |
| N (% female) | 5 (20) | 5 (100) |
| Median age in years (min-max) | 35 (27-53) | 38 (34-50) |
| Disease duration in months | NA | 124 (59-202) |
| Clinical responders, n | NA | 3 |

**Supplementary Table 2:** Used antibodies for this study

| ***Ex vivo*** |  |  |  |
| --- | --- | --- | --- |
| **Marker** | **Clone** | **Fluorescent label** | **Supplier** |
| CCR4 | L291H4 | PE-Cy7 | Biolegend |
| CCR6 | G034E3 | PE | Biolegend |
| CD14 | MφP-9 | APC | BD Biosciences |
| CD243 (MDR1) | UIC2 | APC | Biolegend |
| CD25 | 2A3 | BV605 | BD Biosciences |
| CD3 | SK7 | BV785 | Biolegend |
| CD4 | OKT4 | BV510 | Biolegend |
| CD45RA | HI100 | APC-H7 | BD Biosciences |
| CD8 | SK1 | PerCP-Cy5.5 | BD Biosciences |
| CXCR3 | G025H7 | BV421 | Biolegend |
| VLA-4 | 9F10 | BV711 | BD Biosciences |

| **Gene name** | **Protein name** | **Forward/Reverse** | **5’-3’ sequence** |
| --- | --- | --- | --- |
| *ABCB1* | MDR1 | Forward | GGAAATTTAGAAGATCTGATGTCAAAC |
|  |  | Reverse | CACTGTAATAATAGGCATACCTGGTC |
| *CYP24A1* | CYP24A1 | Forward | GGGGTCTCAAGAAACAGCAC |
|  |  | Reverse | TGCGGAAAATCTTGCCATAC |
| *CSF2* | GM-CSF | Forward | TCTCAGAAATGTTTGACCTCCA |
|  |  | Reverse | GCCCTTGAGCTTGGTGAG |
| *IL17A* | IL-17A | Forward | TGGGAAGACCTCATTGGTGT |
|  |  | Reverse | GGATTTCGTGGGATTGTGAT |
| *IFNG* | IFN-γ | Forward | GGCATTTTGAAGAATTGGAAAG |
|  |  | Reverse | TTTGGATGCTCTGGTCATCTT |
| *VDR* | VDR | Forward | ACCCCTGGGCTCCACTTA |
|  |  | Reverse | GGTCAAAGTCTCCAGGGTCA |

**Supplementary Table 3:** Used primer sets for qPCR
